# Supplementary figures and images for: Impact of electrolyte abnormalities and adverse outcomes in persons with eating disorders: A systematic review protocol
Source: PLoS One. 2024 Aug 8;19(8):e0308000. doi: 10.1371/journal.pone.0308000 (PMC11309401; doi:10.1371/journal.pone.0308000)

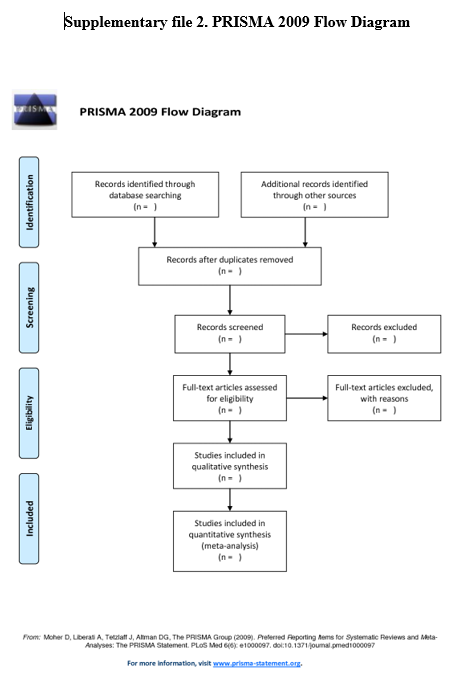

Supplement: S2 File — (PNG) [file pone.0308000.s002.PNG]
