# Supplementary material for: Impact of electrolyte abnormalities and adverse outcomes in persons with eating disorders: A systematic review protocol
Source: PLoS One. 2024 Aug 8;19(8):e0308000. doi: 10.1371/journal.pone.0308000 (PMC11309401; doi:10.1371/journal.pone.0308000)
Supplement: S3 File — (PDF) [file pone.0308000.s003.pdf]

Supplementary file 3

Medline search strategy

1. "feeding and eating disorders"/
2. anorexia nervosa/
3. bulimia nervosa/
4. binge-eating disorder/
5. ((eat* adj3 disorder*) or (binge adj3 eat*)).ti,ab,kf.
6. bulimia.ti,ab,kf.
7. anorexia.ti,ab,kf.
8. or/1-7
9. water-electrolyte imbalance/
10. dehydration/
11. hypercalcemia/
12. Hyperkalemia/
13. hypernatremia/
14. hypocalcemia/
15. hyponatremia/
16. acidosis/ or alkalosis/
17. ((electrolyte adj2 disorder*) or dehydration or hypokelemia or hyperkalemia or metabolic alkalosis or metabolic acidosis or hyponatremia or hypomagnesemia or hypophosphatemia or (water-electrolyte adj2 imbalance)).ti,ab,kf.
18. or/9-17
19. 8 and 18
20. (teen* or adolescent* or youth or adult* or man or men or woman or women or senior* or elderly).ti,ab,kf.
21. 19 and 20
22. limit 19 to ("all adult (19 plus years)" or "adolescent (13 to 18 years)")
23. 21 or 22
24. limit 23 to (english or french)
