# Supplementary material for: Impact of electrolyte abnormalities and adverse outcomes in persons with eating disorders: A systematic review protocol
Source: PLoS One. 2024 Aug 8;19(8):e0308000. doi: 10.1371/journal.pone.0308000 (PMC11309401; doi:10.1371/journal.pone.0308000)
Supplement: S4 File — (PDF) [file pone.0308000.s004.pdf]

Supplementary file 4

Joanna Briggs Institute for Meta-Analysis of Statistics Assessment and Review Instruments

1. JBI critical appraisal checklist for randomized control trials

**Assessor:**	**Date of Appraisal:**	**Record Number:**
**Study Author:**	**Study Title:**	**Study Year:**

Internal Validity		Choice - Comments/Justification	Yes	No	Unclear	N/A
**Bias related to selection and allocation**						
1	**Was true randomization used for assignment of participants to treatment groups?**		☐	☐	☐	☐
2	**Was allocation to treatment groups concealed?**		☐	☐	☐	☐
3	**Were treatment groups similar at the baseline?**		☐	☐	☐	☐
**Bias related to administration of intervention/exposure**						
4	**Were participants blind to treatment assignment?**		☐	☐	☐	☐
5	**Were those delivering the treatment blind to treatment assignment?**		☐	☐	☐	☐
6	**Were treatment groups treated identically other than the intervention of interest?**		☐	☐	☐	☐

Bias related to assessment, detection and measurement of the outcome

7	Were outcome assessors blind to treatment assignment?		Yes	No	Unclear	N/A
	Outcome 1		☐	☐	☐	☐
	Outcome 2		☐	☐	☐	☐
	Outcome 3		☐	☐	☐	☐
	Outcome 4		☐	☐	☐	☐
	Outcome 5		☐	☐	☐	☐
	Outcome 6		☐	☐	☐	☐
	Outcome 7		☐	☐	☐	☐

8	Were outcomes measured in the same way for treatment groups?		Yes	No	Unclear	N/A
	Outcome 1		☐	☐	☐	☐
	Outcome 2		☐	☐	☐	☐
	Outcome 3		☐	☐	☐	☐
	Outcome 4		☐	☐	☐	☐
	Outcome 5		☐	☐	☐	☐
	Outcome 6		☐	☐	☐	☐
	Outcome 7		☐	☐	☐	☐

9	Were outcomes measured in a reliable way		Yes	No	Unclear	N/A
	Outcome 1		☐	☐	☐	☐
	Outcome 2		☐	☐	☐	☐
	Outcome 3		☐	☐	☐	☐
	Outcome 4		☐	☐	☐	☐
	Outcome 5		☐	☐	☐	☐
	Outcome 6		☐	☐	☐	☐
	Outcome 7		☐	☐	☐	☐

Bias related to participant retention

10	Was follow up complete and if not, were differences between groups in terms of their follow up adequately described and analysed?					
	**Outcome 1**		**Yes**	**No**	**Unclear**	**N/A**
	Result 1		☐	☐	☐	☐
	Result 2		☐	☐	☐	☐
	Result 3		☐	☐	☐	☐
	**Outcome 2**		**Yes**	**No**	**Unclear**	**N/A**

Result 1		☐	☐	☐	☐
Result 2		☐	☐	☐	☐
Result 3		☐	☐	☐	☐
**Outcome 3**		**Yes**	**No**	**Unclear**	**N/A**
Result 1		☐	☐	☐	☐
Result 2		☐	☐	☐	☐
Result 3		☐	☐	☐	☐
**Outcome 4**		**Yes**	**No**	**Unclear**	**N/A**
Result 1		☐	☐	☐	☐
Result 2		☐	☐	☐	☐
Result 3		☐	☐	☐	☐
**Outcome 5**		**Yes**	**No**	**Unclear**	**N/A**
Result 1		☐	☐	☐	☐
Result 2		☐	☐	☐	☐
Result 3		☐	☐	☐	☐
**Outcome 6**		**Yes**	**No**	**Unclear**	**N/A**
Result 1		☐	☐	☐	☐
Result 2		☐	☐	☐	☐
Result 3		☐	☐	☐	☐

Outcome 7		Yes	No	Unclear	N/A
Result 1		☐	☐	☐	☐
Result 2		☐	☐	☐	☐
Result 3		☐	☐	☐	☐

Statistical Conclusion Validity

11	**Were participants analysed in the groups to which they were randomized?**					
	**Outcome 1**		**Yes**	**No**	**Unclear**	**N/A**
	Result 1		☐	☐	☐	☐
	Result 2		☐	☐	☐	☐
	Result 3		☐	☐	☐	☐
	**Outcome 2**		**Yes**	**No**	**Unclear**	**N/A**
	Result 1		☐	☐	☐	☐
	Result 2		☐	☐	☐	☐
	Result 3		☐	☐	☐	☐
	**Outcome 3**		**Yes**	**No**	**Unclear**	**N/A**
	Result 1		☐	☐	☐	☐
	Result 2		☐	☐	☐	☐

Result 3		☐	☐	☐	☐
**Outcome 4**		**Yes**	**No**	**Unclear**	**N/A**
Result 1		☐	☐	☐	☐
Result 2		☐	☐	☐	☐
Result 3		☐	☐	☐	☐
**Outcome 5**		**Yes**	**No**	**Unclear**	**N/A**
Result 1		☐	☐	☐	☐
Result 2		☐	☐	☐	☐
Result 3		☐	☐	☐	☐
**Outcome 6**		**Yes**	**No**	**Unclear**	**N/A**
Result 1		☐	☐	☐	☐
Result 2		☐	☐	☐	☐
Result 3		☐	☐	☐	☐
**Outcome 7**		**Yes**	**No**	**Unclear**	**N/A**
Result 1		☐	☐	☐	☐
Result 2		☐	☐	☐	☐
Result 3		☐	☐	☐	☐

12	Was appropriate statistical analysis used?					
	**Outcome 1**		**Yes**	**No**	**Unclear**	**N/A**
	Result 1		☐	☐	☐	☐
	Result 2		☐	☐	☐	☐
	Result 3		☐	☐	☐	☐
	**Outcome 2**		**Yes**	**No**	**Unclear**	**N/A**
	Result 1		☐	☐	☐	☐
	Result 2		☐	☐	☐	☐
	Result 3		☐	☐	☐	☐
	**Outcome 3**		**Yes**	**No**	**Unclear**	**N/A**
	Result 1		☐	☐	☐	☐
	Result 2		☐	☐	☐	☐
	Result 3		☐	☐	☐	☐
	**Outcome 4**		**Yes**	**No**	**Unclear**	**N/A**
	Result 1		☐	☐	☐	☐
	Result 2		☐	☐	☐	☐
	Result 3		☐	☐	☐	☐
	**Outcome 5**		**Yes**	**No**	**Unclear**	**N/A**

		Yes	No	Unclear	N/A
Result 1		☐	☐	☐	☐
Result 2		☐	☐	☐	☐
Result 3		☐	☐	☐	☐
**Outcome 6**		**Yes**	**No**	**Unclear**	**N/A**
Result 1		☐	☐	☐	☐
Result 2		☐	☐	☐	☐
Result 3		☐	☐	☐	☐
**Outcome 7**		**Yes**	**No**	**Unclear**	**N/A**
Result 1		☐	☐	☐	☐
Result 2		☐	☐	☐	☐
Result 3		☐	☐	☐	☐

			Yes	No	Unclear	N/A
13	**Was the trial design appropriate and any deviations from the standard RCT design (individual randomization, parallel groups) accounted for in the conduct and analysis of the trial?**		☐	☐	☐	☐

Overall appraisal: **Include:** ☐ **Exclude:** ☐ **Seek Further Info:** ☐

Comments:

Table 3 – The JBI Critical Appraisal Tool for RCTs

2. JBI critical appraisal checklist for quasi-experimental s (non-randomized experimental) studies

Reviewer_____ ________________________________ Date______________________________

Author_______________________________________ Year_________ Record Number_________

	Yes	No	Unclear	Not applicable
1. Is it clear in the study what is the 'cause' and what is the 'effect' (i.e. there is no confusion about which variable comes first)?	☐	☐	☐	☐
2. Were the participants included in any comparisons similar?	☐	☐	☐	☐
3. Were the participants included in any comparisons receiving similar treatment/care, other than the exposure or intervention of interest?	☐	☐	☐	☐
4. Was there a control group?	☐	☐	☐	☐
5. Were there multiple measurements of the outcome both pre and post the intervention/exposure?	☐	☐	☐	☐
6. Was follow up complete and if not, were differences between groups in terms of their follow up adequately described and analyzed?	☐	☐	☐	☐
7. Were the outcomes of participants included in any comparisons measured in the same way?	☐	☐	☐	☐
8. Were outcomes measured in a reliable way?	☐	☐	☐	☐
9. Was appropriate statistical analysis used?	☐	☐	☐	☐

Overall appraisal: Include ☐ Exclude ☐ Seek further info ☐

Comments (Including reason for exclusion)

____
