# Supplementary material for: Impact of electrolyte abnormalities and adverse outcomes in persons with eating disorders: A systematic review protocol
Source: PLoS One. 2024 Aug 8;19(8):e0308000. doi: 10.1371/journal.pone.0308000 (PMC11309401; doi:10.1371/journal.pone.0308000)
Supplement: S5 File — (PDF) [file pone.0308000.s005.pdf]

Supplementary file 5

JBI Data Extraction Form for Experimental / Observational Studies

Reviewer ________________________ Date ________________________

Author ________________________ Year ________________________

Journal ________________________ Record Number ________________

Study Method

RCT	☐	Quasi-RCT	☐	Longitudinal	☐
Retrospective	☐	Observational	☐	Other	☐

Participants

Setting ________________________

Population ________________________

Sample size

Group A ________________ Group B ________________

Interventions

Intervention A ________________________

Intervention B ________________________

Authors Conclusions:

Reviewers Conclusions:

Study results

Dichotomous data

Outcome	Intervention ( ) number / total number	Intervention ( ) number / total number

Continuous data

Outcome	Intervention ( ) number / total number	Intervention ( ) number / total number

Source: JBI Database of Systematic Reviews and Implementation Reports15(7):1835-1849, July 2017.
