# Supplementary material for: Impact of electrolyte abnormalities and adverse outcomes in persons with eating disorders: A systematic review protocol
Source: PLoS One. 2024 Aug 8;19(8):e0308000. doi: 10.1371/journal.pone.0308000 (PMC11309401; doi:10.1371/journal.pone.0308000)
Supplement: Attachment — Submitted filename: Response to Reviewers.docx [file pone.0308000.s007.docx]

July 13, 2024

**Manuscript reference #:** PONE-D-24-15566R1

**Title:** Impact of electrolyte abnormalities and adverse outcomes in persons with eating disorders: a systematic review protocol

RE: PLOS ONE Decision: Revision required [PONE-D-24-15566R1] - [EMID:4c1da07e9e1ed7f8]

Dear Dr. Muhammad Shahzad Aslam,

We are very grateful for the comments and for the opportunity to revise our manuscript further. We believe we have addressed all comments and are happy to submit a revised version of the manuscript. Please see below a point-by-point response to the comments.

Sincerely,

Amos Buh, PhD, FRSPH 
Corresponding Author 

**Response to Reviewers’ reports**

Authors should check the supplementary file 1 which seems to be filled for a different study with the headline "Effective educational interventions for the promotion of sexual and reproductive health and rights for school-age children in low and middle-income countries: A systematic review protocol".

[Response:](https://plos.org/protocols?utm_medium=editorial-email&utm_source=authorletters&utm_campaign=protocols) We greatly appreciate this observation. We made an error with the headline while filling this file; the error has been corrected and the headline now reads “Impact of electrolyte abnormalities and adverse outcomes in persons with eating disorders: a systematic review protocol”. Thank you.

It would be really very interesting and novel review paper. Hope to open the new avenue of research in future on the same topic. Really exciting to see as soon in published form.

[Response:](https://plos.org/protocols?utm_medium=editorial-email&utm_source=authorletters&utm_campaign=protocols) Thank you so much for appraising our manuscript. We hope our work on this topic will inspire future research on this same topic. Once we complete the review, we will submit the final manuscript for publication so that everyone can have access to our findings.

Categorically explain the aim, research question, background, study and methodology of the study. Upon on review the supplementary attached documents which more clear the novelty and comprehension of the stud.

[Response:](https://plos.org/protocols?utm_medium=editorial-email&utm_source=authorletters&utm_campaign=protocols) We are deeply grateful to you for taking a keen look on our manuscript and appreciating its clarity from the aim through the research question, background and study methods to the supplementary documents. Thank you.

However, minor changes in background chapter required to add more evidence-based studies on biological explanation how electrolytes imbalance in individual with eating disorder cause health adverse outcomes with sign and symptoms. Categorically explain the complications in ED as outcome of the electrolytes disorders.

[Response: Thank you for this suggestion. We have expanded our background section (particularly the last paragraph of the background) to include additional text regarding the existing literature on how electrolyte disorders may lead to adverse health outcomes (pages 3-4, lines 77-93): “The severity and type of electrolyte abnormalities are dependent on the mode, and frequency of the behaviors of the eating disorder [17]. Some signs and symptoms of electrolyte imbalances in people with eating disorders include constipation, nausea and vomiting, fatigue, heart palpitations or arrhythmias, muscle weakness, cramps, numbness, polyuria, headache, confusion, restlessness and irritability [18]. While complications of eating disorders range from minor to fatal, every organ system can be affected with problems ranging from pancytopenia to diffuse myalgias with muscle breakdown [19]. In fact, eating disorders can impact neurological, cardiac, muscle, gastrointestinal, endocrine and renal functions [11, 18, 20]. However, it has been documented that electrolyte abnormalities are associated with a host of serious adverse clinical outcomes [12]. For instance, derangements in potassium, magnesium, and calcium levels predispose to cardiac arrhythmias and sudden cardiac death [18]. Derangements in sodium and phosphate levels can result in generalized weakness, cognitive impairment, and seizures [19, 20]. Electrolyte disturbances can also have detrimental effects on kidney, gastrointestinal and bone health [21 - 23]. Nonetheless, there is little or no information on reviews that have comprehensively assessed the impact of electrolyte abnormalities and adverse outcomes resulting from people with eating disorders.”](https://plos.org/protocols?utm_medium=editorial-email&utm_source=authorletters&utm_campaign=protocols)
